# Supplementary material for: Social support and professional networks of nurses and nursing technicians in coping with Covid-19: A sectional study in two Brazilian cities
Source: PLoS One. 2023 Jan 23;18(1):e0280357. doi: 10.1371/journal.pone.0280357 (PMC9870100; doi:10.1371/journal.pone.0280357)
Supplement: S1 File — (DOCX) [file pone.0280357.s001.docx]

FULL NAME (IF YOU PREFER, PUT INITIALS): * Your answer

AGE: * Your answer

SEX: *

• FEMININE

• MASCULINE

EDUCATION DEGREE: *

• INCOMPLETE FUNDAMENTAL

• FUNDAMENTAL COMPLETE

• INCOMPLETE AVERAGE

• COMPLETE AVERAGE

•INCOMPLETE HIGHER

• GRADUATED

• INCOMPLETE SPECIALIZATION

• FULL SPECIALIZATION

• INCOMPLETE RESIDENCE

• COMPLETE RESIDENCE

• INCOMPLETE MASTER'S

• COMPLETE MASTER'S

• INCOMPLETE DOCTORATE

• FULL DOCTORATE

•POST DOCTORAL

ARE YOU A HEALTH PROFESSIONAL OF THE UNIT OR VOLUNTEER? *

• UNIT HEALTH PROFESSIONAL

• VOLUNTEER

IF YOU ARE A HEALTH PROFESSIONAL AT THE UNIT, WHAT IS YOUR PROFESSIONAL CATEGORY: *

•NURSING ASSISTANT

• NURSING TECHNICIAN

•NURSE

•DOCTOR

• LABORATORY TECHNICIAN OR ASSISTANT

•DENTIST

•PHYSICAL THERAPIST

•NUTRITIONIST

• SOCIAL ASSISTANT

• OTHER PROFESSIONAL CATEGORY

IF YOU ARE A VOLUNTEER, WHAT IS YOUR PROFESSIONAL CATEGORY: *

•NURSING ASSISTANT

• NURSING TECHNICIAN

•NURSE

•DOCTOR

• LABORATORY TECHNICIAN OR ASSISTANT

•DENTIST

•PHYSICAL THERAPIST

•NUTRITIONIST

• SOCIAL ASSISTANT

• OTHER PROFESSIONAL CATEGORY

HOW LONG DO YOU WORK, IF YOU ARE A HEALTH PROFESSIONAL AT THE UNIT? *

• < 01 YEAR

• 2 TO 3 YEARS

• 3 TO 4 YEARS

• 4 TO 5 YEARS

• 5 YEARS

• I AM A VOLUNTEER

IF VOLUNTEER, WHAT IS YOUR TRAINING TIME? *

• < 01 YEAR

• 2 TO 3 YEARS

• 3 TO 4 YEARS

• 4 TO 5 YEARS

• 5 YEARS

RECEIVED TRAINING TO USE PERSONAL PROTECTIVE EQUIPMENT

AMONG THE ITEMS BELOW, WHICH ARE NECESSARY FOR PROFESSIONALS WHO ACT DIRECTLY IN THE CARE OF A SUSPECTED OR CONFIRMED PATIENT FOR CODIV-19 (WEARING AND DESPARING)? *

• YES

• NO

• I MISSED THE SCHEDULED TRAINING

CONSIDER THAT THE TRAINING RECEIVED WAS *

• ENOUGH

• PARTIALLY ENOUGH

• INSUFFICIENT

AMONG THE ITEMS BELOW, WHICH ARE NECESSARY FOR PROFESSIONALS WHO ACT DIRECTLY IN THE CARE OF A SUSPECTED OR CONFIRMED PATIENT FOR CODIV-19?

• COVER / APRON

• N95 MASK

• SURGICAL MASK

•PROTECTIVE GOGGLES

• CAP

• PROPÉ

• STERILE GLOVE

• PROCEDURE GLOVE

• FACE PROTECTOR

DO YOU HAVE DOUBTS ABOUT THE PROPER USE OF PERSONAL PROTECTIVE EQUIPMENT? *

• YES, MANY DOUBTS

YES, FEW DOUBTS

I HAVE NO DOUBTS

BRIEFLY EXPLAIN YOUR QUESTION

Your answer

• THE HEALTH UNIT IN WHICH IT OPERATES HAS AVAILABLE PPE IN QUANTITY

IF THE ANSWER TO THE PREVIOUS ITEM HAS BEEN "RARELY OR NEVER", CHECK WHICH

ENOUGH TO CHANGE DURING WORK SHIFT? *

• YES, PPE ARE ALWAYS AVAILABLE IN ROUTINE

• I ALMOST ALWAYS HAVE PPE TO WORK

• I RARELY HAVE PPE TO WORK

• I NEVER HAVE PPE TO WORK

ITEMS HAVE NOT BEEN PROVIDED: *

• COVER / APRON

• N95 MASK

• SURGICAL MASK

•PROTECTIVE GOGGLES

• CAP

• FEET COVER

• STERILE GLOVE

• PROCEDURE GLOVE

• FACE PROTECTOR

DO YOU CONSIDER THAT THE QUALITY OF THE PPE PROVIDED IS ADEQUATE? *

• YES, FOR ALL PPE

• YES, FOR SOME PPE

• NO, THE PPE ARE OF LOW QUALITY

SAY WHICH SUPPLIED PPE YOU EVALUATE TO BE LOW QUALITY *

• COVER / APRON

• N95 MASK or SIMILAR

• SURGICAL MASK

•PROTECTIVE GOGGLES

• CAP

• FEET COVER

• STERILE GLOVE

• PROCEDURE GLOVE

• FACE PROTECTOR

CONSIDERS THE STRUCTURE AND THE FLOW AND SERVICES TO PATIENTS UNDER SUSPECTED OR

CONFIRMED FOR ADEQUATE CODIV-19: *

• YES

• NO

• PARTIALLY SUITABLE

IF YOU MARKED "NO" OR "PARTIALLY", EXPLAIN WHY

CHECK UP TO 05 PROFESSIONALS WHO, IN THEIR WORK, ARE OUTSTANDING IN SERVICE TO HEALTH PROFESSIONALS WITH SUSPECTED COVID-19 *

Your answer

• ADMINISTRATOR

• CHIEF NURSE

• TEAM NURSE

• NURSING ASSISTANT OR TECHNICIAN

• GENERAL PHYSICIAN

• ASSISTANT OR LABORATORY TECHNICIAN

• INFECTOLOGIST DOCTOR

• DOCTORS WITH OTHER SPECIALTIES

• ASSISTANT OR PHARMACY TECHNICIAN

•ADMINISTRATIVE ASSISTANT

• MULTIPROFESSIONAL TRAINING TEAM

•PHARMACEUTICAL

• SOCIAL ASSISTANT

•PHYSICAL THERAPIST

•NUTRITIONIST

• COMMUNITY HEALTH AGENT

•DRIVER

• MUNICIPAL OR STATE MANAGER

• BIOLOGIST

CHECK UP TO 05 PROFESSIONALS WHO, IN THEIR WORK, ARE OUTSTANDING TO GUARANTEE SUPPLY OF PPE FOR NURSING *

• ADMINISTRATOR

• CHIEF NURSE

• TEAM NURSE

• NURSING ASSISTANT OR TECHNICIAN

• GENERALIST DOCTOR

• INFECTOLOGIST DOCTOR

• PHYSICIAN OF ANOTHER SPECIALTY

• ASSISTANT OR PHARMACY TECHNICIAN

• ASSISTANT OR ADMINISTRATIVE TECHNICIAN

• MULTIPROFESSIONAL TRAINING TEAM

•PHARMACEUTICAL

• SOCIAL ASSISTANT

•PHYSICAL THERAPIST

•NUTRITIONIST

• COMMUNITY HEALTH AGENT

•DRIVER

• MUNICIPAL OR STATE MANAGER

• BIOLOGIST
