# Supplementary material for: Social support and professional networks of nurses and nursing technicians in coping with Covid-19: A sectional study in two Brazilian cities
Source: PLoS One. 2023 Jan 23;18(1):e0280357. doi: 10.1371/journal.pone.0280357 (PMC9870100; doi:10.1371/journal.pone.0280357)
Supplement: Attachment — Submitted filename: Author responses 21 March 2022.docx [file pone.0280357.s006.docx]

*21 March 2022*

*Comments with author responses for*

*manuscript* **Social support and professional networks of nurses and nursing technicians in coping with Covid-19: a sectional study in two Brazilian cities**

*DAVID et. al.*

As authors, we are grateful to the Editor and the Peer reviewers for their careful review of our manuscript and detailed comments. This allowed us to carry out a major review of the manuscript. We attempted to address each question, comment and suggestion by the reviewers. Below is a bulleted version of our actions.

Reviewer 1
1. The authors could double check the structural and grammar aspects in the following sentences:

“Networks can, in terms of homogeneity and reciprocity between their actors, be classified as type-1 (total network) or mode-2.”

and

“It’s an observational and cross-sectional research, based on the social network analysis methodology, using an online data

collection instrument.”

The entire text was submitted to English proofreading.

2.  The authors state that

“The study of social networks began in the 1930s within the scope of the social sciences.”

and

“The concept of social support is widely disseminated and studied by the scientific community.”

These statements should be referenced.

We included the corresponding references.

3. The authors should be clear to all readers, not only those from Social Science. Therefore, I suggest that they introduce the

nomenclature used by social scientists, physicists, and mathematicians in the studying of social networks at the beginning and

adopt only one throughout the manuscript. For example, the authors state that

“The former exists when the actors (nodes) who mention other actors (also called links or alters) are part of the same network, in a

square matrix.”

Links are edges (ties) for Physicists. The authors should avoid this kind of problem.

We used the most common nomenclature adopted in Social Network Analysis, and attempted to make it more intelligible.

4. It is imperative that the authors include a didactic figure explaining their model precisely. Further, I think the authors could emphasize that their model is a bipartite network at the beginning, not only at the ending.

We included a didactic frame summarizing the research model (Frame 1).

5. I think the Social Network Analysis performed was quite superficial. There are no histograms or scatter plots of any kind, there is only 1 table and 4 figures (which could be combined into a single panel). Further, the quality of all figures is very low. The authors should improve their analysis.

This SNA study was based on egocentric networks. Sociograms are usually the only graphic representation employed.

The four sociograms were organized in one unique figure (Figure 1). Figure met the author’s guide requirements regarding the quality of images.

6. Why are the results not biased due to the chosen sample of participants (only nurses and nursing technicians)? For example, if the questionnaire was applied to janitors, I believe the result would be the janitors care more about the health of other janitors. The authors must clarify this point.

The research was focused on social relationships between each professional (ego) and other professionals according to their position/category. Each respondent could choose any one of the presented categories. A paragraph was inserted in the final part of the Discussion section, where we comment on this potential bias limitation.

Reviewer 2

General comment: The manuscript would benefit from editorial review, as there are incomplete or confusing sentences throughout

the manuscript.

Introduction: Please make clear the gap being addressed by this study as there has been considerable information published about the nurse’s experience during COVID. In addition, 2nd line of 1st paragraph – not certain “control” is the most appropriate word.

We included the lack of studies of this type during the pandemic, and changed the verb "control".

Research question: Seems focus was on two types of nurse: nurses with baccalaureate and nurse technicians. Suggest rewording

RQ. “ What are the social networks of nurses and nurse technicians during COVID?” or something to make it clear you are simply

describing social networks of two different nurse types. If you intended to compare, then that should be included in the RQ.

We changed the RQ.

Social Networks: 3rd paragraph in this section: one sentence paragraph – not certain the purpose of this information. Mention is made of SNA studies but no references are provided contributing to a lack of clarity.

We included the corresponding references.

Social support at work: Please clarify who provides the assistance and protection to professionals. Perhaps this information could

be combined with the previous discussion on social support – with greater emphasis on findings. Also please consider including

more than one citation when using phrases such as “several studies.”

We merged the two topics (social networks and social support) into one and we corrected the phrases.

Methods: Believe you can use subheadings – which would be very helpful to the reader. Design: suggest using cross-sectional onlyas that design is one type of observational design. Please include more discussion about the social network analysis methodology. In fact, consider a subheading of data analysis – this would be the place to discuss SNA in greater depth. Describe how the list was

developed, based on the literature?? Consider a subheading for participantsthat provides inclusion criteria, desired number of participants, rationale for two different clinical settings and sites, who approached them to invite them, where were they approached, etc. Were any demographic data collected about the participants? If so please indicate and then describe under findings.

Including a subheading called “data analysis” would strengthen the manuscript and could include the discussion of centrality metrics. Make clear these metrics are the key to SNA. Also include the type of analysis performed – descriptive statistics? Research Ethics: The statement that is made implies IRB was obtained for a previous study, not this one. Please clarify and if

possible, provide the IRB reference number.

The Methods section was expanded and reorganized. We also added detailed information on the statistical methods.

Results: Please note phraseology in the first sentence. Should be reordered as something is presented in a table, not a table presenting something – this is a common mistake . However, please consider revising the tables to reflect a clear purpose. Provide sample number- consider only including the top five and bottom five social networks. Somehow, the numbers included in the table

need to discussed better. For example, Table 1, Nurse has 0.905 for Degree – but it is not clear what this number means. Is it the mean, total score? Some goes for Table 2. In addition, please be consistent in presenting findings: Nurses first, followed by nurse technicians. Note that differences are references when discussing proximity scores but differences was not mentioned in the research question. Consider a paragraph at the end of findings that clearly states the conclusions drawn from the findings. This could be the first paragraph of Discussion instead – what is important is that the reader needs to be clear about what conclusions can be drawn for these data. For example, is it possible nurses and nurse technicians have similar social networks? 3rd

paragraph – support the first sentence with citations. Moreover, if social support has been addressed in Brazilian literature, make clear the contribution being made by this study.

We improved the phraseology and provided the English language proofreading of the manuscript. The centrality measures results are commented both in the Results and Discussion sections.

Conclusion: The term “informal” is introduced – do not recall seeing the term earlier in the paper. Was this the focus of the study – if so, please make clear from the outset.

Please address limitations associated with the study and make clear the implications. What do findings mean for nurses and technicians?

References: Several references are in a language other the English, making retrieval and understanding of the work inaccessible to

an international audience. Please consider replacing with other references.

Figures – please provide legends to help the reader understand information being presented.

We included a comment about "informal relationships" in the Discussion section. English language was improved and we provided figures’ captions.

We remain at your disposal for further clarifications.

**Helena Maria Scherlowski Leal David**

Corresponding Author
