# Supplementary material for: Social support and professional networks of nurses and nursing technicians in coping with Covid-19: A sectional study in two Brazilian cities
Source: PLoS One. 2023 Jan 23;18(1):e0280357. doi: 10.1371/journal.pone.0280357 (PMC9870100; doi:10.1371/journal.pone.0280357)
Supplement: Attachment — Submitted filename: Response to the Reviewer 2.docx [file pone.0280357.s007.docx]

Response to the Reviewer 2

1. Page 6; last paragraph: who provides the social support to professionals?

R – We provided the requested information.

2. Network data and collection: Information in this section needs to be re-orgranized. Suggest subheadings of Setting and Sample - two separate headings. Combine all the sample information into one section and make clear whether a desired number of participants was needed. Expand on how the sample was recruited - blast email to employees? word of mouth?

R – The section was reorganized. We added the requested information about sample size and recruiting.

3. Instrument: Please provide more information about the questions asked - did anyone review them for validity?

R – We added information about validity.

4. Page 10: Human Research Ethics Committee: Information presented here makes this reviewer question whether the current study is secondary analysis of another larger study - please clarify.

R – We clarified this item.
